# Emerging Roles for NlpE as a Sensor for Lipoprotein Maturation and Transport to the Outer Membrane in Escherichia coli

**DOI:** 10.1128/mBio.01302-19

**Published:** 2019-06-25

**Authors:** Brent W. Simpson, M. Stephen Trent

**Affiliations:** a Department of Infectious Diseases, College of Veterinary Medicine, University of Georgia, Athens, Georgia, USA; b Center for Vaccines and Immunology, College of Veterinary Medicine, University of Georgia, Athens, Georgia, USA; c Department of Microbiology, Franklin College of Arts and Sciences, University of Georgia, Athens, Georgia, USA

**Keywords:** CpxAR, Lol, NlpE, Rcs, copper, lipoproteins, outer membrane

## Abstract

Outer membrane biogenesis is a complex process for Gram-negative bacteria as the components are synthesized in the cytoplasm or at the inner membrane and then transported to the outer membrane. Stress pathways monitor and respond to problems encountered in assembling the outer membrane. The two-component system CpxAR was recently reported to be a stress pathway for transport of lipoproteins to the outer membrane, but it was unclear how this stress is sensed. May et al. [K. L. May, K. M. Lehman, A. M. Mitchell, and M. Grabowicz, mBio 10(3):e00618-19, 2019, https://doi.org/10.1128/mBio.00618-19] determined that an outer membrane lipoprotein, NlpE, is the sensor for lipoprotein biogenesis stress. The group demonstrated that CpxAR is activated by the N-terminal domain of NlpE when the lipoprotein accumulates at the inner membrane. Further, this work resolved a previously debated role for NlpE in sensing copper stress; copper was shown to inhibit acylation of lipoproteins, preventing them from being transported to the outer membrane.

## Abstract (precis)

Outer membrane biogenesis is a complex process for Gram-negative bacteria as the components are synthesized in the cytoplasm or at the inner membrane and then transported to the outer membrane. Stress pathways monitor and respond to problems encountered in assembling the outer membrane. The two-component system CpxAR was recently reported to be a stress pathway for transport of lipoproteins to the outer membrane, but it was unclear how this stress is sensed. May et al. [K. L. May, K. M.

## COMMENTARY

Gram-negative bacteria build a distinctive diderm (two lipid bilayers) cell envelope that provides unique advantageous properties, including particular resistance to permeation of toxic compounds and antibiotics (1). The stringent permeability characteristics of the Gram-negative cell envelope are imparted largely by an asymmetric outer membrane (OM) with glycerophospholipids in the inner leaflet and lipopolysaccharide (LPS) in the outer leaflet (1). However, building an OM comes with unique challenges, as the components are synthesized in the cytoplasm or at the inner membrane (IM) and then transported across the IM and periplasm. OM biogenesis is further complicated by the lack of ATP or other energy sources in the periplasm. As such, transenvelope assembly complexes exist for delivering the major OM components: Lpt for transport of LPS (2), Bam and periplasmic chaperones for assembly of OM beta-barrel proteins (OMPs) (3), and Lol for transport of OM lipoproteins (4). Further, assembly of the OM requires proper coordination between OM assembly complexes. To monitor and adjust each of the OM assembly processes, Gram negatives have evolved multiple stress responses. While dedicated stress responses for assessing LPS and OMP biogenesis have been described previously (5, 6), how cells respond to defects in OM lipoprotein biogenesis has remained unclear. May et al. (7) provide new evidence that an OM lipoprotein, NlpE, and the two-component system CpxAR serve as a stress response for defects in lipoprotein maturation and transport to the OM.

**FIG 1:**
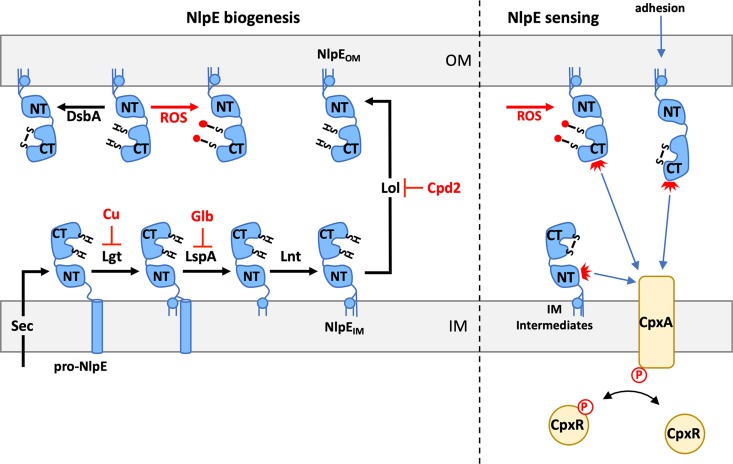
NlpE is a multipurpose sensor for lipoprotein biogenesis, disulfide bond formation, and surface adhesion. (Left) Biogenesis of the outer membrane (OM) lipoprotein NlpE (blue) begins with transport across the inner membrane (IM) by the SecYEG complex. The prolipoprotein, pro-NlpE, is then processed in an ordered manner by Lgt, which transfers a diacylglycerol to the +1 Cys residue, LspA, which cleaves off the signal peptidase, and Lnt, which transfers an acyl-chain to the amino terminus of +1 Cys. NlpE is finally transported to the OM by LolABCDE or by a second LolCDE alternative pathway. NlpE contains two Cys residues in the N-terminal domain (labeled “NT”; Cys not shown) and two Cys residues in the C-terminal domain (labeled “CT”; sulfurs of Cys residues shown) that form a disulfide bonded pair within each domain catalyzed by DsbA. DsbA-mediated disulfide bond formation is shown at the OM for simplicity but can occur at any step of NlpE biogenesis. Compounds that inhibit NlpE biogenesis are shown in red: copper (Cu) modifies the thiol of the +1 Cys, preventing Lgt catalysis; globomycin (Glb) inhibits signal peptidase LspA; compound 2 (Cpd2) inhibits transport to the OM; and reactive oxygen species (ROS) can modify Cys residues, preventing disulfide bond formation (ROS-modified Cys residues are shown as bonding to red circles). (Right) Trapping of NlpE at the IM, such as when lipoprotein biogenesis is inhibited by Cu and Cpd2, leads to activation of the CpxAR two-component system. Activation of sensor kinase CpxA leads to phosphorylation of the response regulator, CpxR. Phosphorylated CpxR in turn regulates the Cpx regulon. CpxA activation by IM-localized NlpE occurs through the NT domain. The CT domain of NlpE can activate CpxA either in response to the oxidation state of the CT Cys residues or when adhesion of the cell to hydrophobic surfaces causes conformational changes in NlpE.

Lipoprotein biogenesis (shown in Fig. 1) begins with transport across the IM by the SecYEG complex, resulting in a prolipoprotein with an N-terminal signal peptide (4). A lipobox sequence at the end of the signal peptide defines the +1 Cys that becomes the first amino acid of the mature form of the lipoprotein (4). This +1 Cys is modified with a thioester-linked diacylglycerol, the signal peptide is cleaved, and, finally, the +1 Cys is modified with an amide-linked fatty acid by the enzymes Lgt, LspA, and Lnt in that order (4). Finally, lipoproteins destined for the OM are transported across the cell envelope by either the traditional LolABCDE transport complex or a recently identified second pathway that requires LolCDE but does not require LolAB (4). Maturation and transport of OM lipoproteins are essential processes because each of the complexes that assemble the OM (Lpt, Bam, and Lol) contains at least one OM lipoprotein (2–4). Thus, it would be advantageous for Gram-negative bacteria to monitor and respond to problems in lipoprotein biogenesis.

Studying the cellular response to lipoprotein biogenesis inhibition in Escherichia coli has traditionally been difficult. It was previously noted that accumulation of the abundant OM lipoprotein Lpp at the IM is toxic, due to improper cross-linking of IM-localized Lpp to the peptidoglycan layer (8). Further, two stress response pathways, Rcs and Cpx, are activated upon inhibition of lipoprotein biogenesis (9). However, activation of the Rcs stress response is toxic under these conditions due to buildup of an Rcs-regulated lipoprotein, OsmB (9), suggesting that Rcs has not evolved to alleviate defects in lipoprotein biogenesis. Meanwhile, activation of Cpx is beneficial to mutants with defective lipoprotein biogenesis (9), supporting the idea of its role in responding to this sort of stress. Still, how CpxAR senses lipoprotein biogenesis defects was not known.

May et al. overcame the problems represented by these complexities in studying lipoprotein biogenesis stress by utilizing E. coli strains carrying an *lpp* (Δ*K58*) allele that encodes a variant incapable of cross-linking to peptidoglycan and a null allele of *osmB* that prevents Rcs toxicity without affecting the rest of the Rcs response. The group then utilized a LolB depletion system that they had previously designed to extensively reduce lipoprotein transport to the OM (9). Upon depletion of LolB, E. coli cells are fully dependent on a second lipoprotein transport pathway recently discovered by Grabowicz and Silhavy (9) that is still being unraveled. Under these conditions, CpxAR activation is also critical for viability to deal with the stress of reduced transport (9).

Two lipoproteins, NlpE and YafY, had previously been shown to activate CpxAR, making them likely candidates for sensors of lipoprotein biogenesis (10, 11). NlpE had been implicated in sensing cell adhesion to hydrophobic surfaces and activating CpxAR (11), while YafY did not have a known function. May et al. found that NlpE activated CpxAR only when LolB was depleted. Since NlpE has two domains (12), i.e., the N-terminal and C-terminal domains, the authors next tested which domain of NlpE activated CpxAR during LolB depletion. The C-terminal domain is thought to be responsible for activation of CpxAR during surface adhesion (12). Conversely, May et al. found that the N-terminal domain was sufficient for Cpx activation when LolB was depleted. Further, by pretreating cells with the protein synthesis inhibitor kasugamycin to ensure that all of the NlpE was fully transported to the OM, the authors demonstrated that new NlpE synthesis is required to sense lipoprotein biogenesis defects and that the existing population of OM-localized NlpE is blind to this stress. Therefore, sensing must occur when NlpE accumulates at the IM. These findings agree with recent work by Delhaye et al. (13); Delhaye and colleagues instead performed experiments with overexpression of a dominant-negative *lolA* allele (which blocks lipoprotein transport to the OM) and found that the N-terminal domain of NlpE was critical for activating CpxAR under these conditions. Parallel and independent findings reported by these two groups from experiments that employed strong bacterial-genetic approaches bolster the idea of a role of NlpE/CpxAR in a lipoprotein biogenesis stress pathway.

To further augment the idea of a role of NlpE in sensing lipoprotein biogenesis defects, May et al. also tested if CpxAR and NlpE provide protection against a LolCDE inhibitor called compound 2 (14) and against an LspA inhibitor, globomycin (15). A Δ*cpxR* mutant was highly sensitive to both inhibitors, while a Δ*nlpE* mutant showed increased sensitivity only to compound 2. These findings indicated that inhibition of LolCDE by compound 2 led to NlpE-dependent activation of CpxAR whereas globomycin treatment activated CpxAR in an NlpE-independent manner. In addition to inhibiting LspA, globomycin has been suggested to have a second activity because globomycin is bactericidal against Mycobacterium tuberculosis despite the fact that LspA is not essential for viability in this bacterium (16). A second activity of globomycin could explain how it activates CpxAR in an NlpE-independent manner.

**Dissecting the role of NlpE in copper sensing.** Finally, May et al. sought to reconcile previous conflicting reports on whether NlpE activates CpxAR in response to copper toxicity (17, 18) and whether such a response was linked to its role in sensing lipoprotein biogenesis. They found that NlpE, specifically, its N-terminal domain, was critical for CpxAR activation and viability under conditions of copper toxicity. Copper reacts with thiol groups of Cys residues, and NlpE contains five Cys residues, including one that is acylated and a disulfide-bonded pair in the N-terminal domain and another in the C-terminal domain, suggesting that modification of one of these Cys could be sensed. May et al. found that disrupting the N-terminal disulfide bond of NlpE by substituting the two Cys residues with Ser did not activate Cpx, indicating that copper sensing was not a consequence of modifying these Cys residues and preventing disulfide bond formation. If copper were modifying the thiol of the +1 Cys of NlpE, then this would prevent the first step of lipoprotein maturation wherein a diacylglycerol is transferred to the thiol of the +1 Cys by Lgt (4). This would result in accumulation of IM-trapped lipoprotein intermediates because the downstream steps depend on Lgt (4). May et al. demonstrated that copper stress indeed resulted in accumulation of the same lipoprotein intermediates as inhibition of Lgt. Since the authors had seen that the N-terminal domain was sufficient to activate CpxAR in response to copper, they did not test disruption of the C-terminal domain disulfide bond. However, it is worth noting that Delhaye et al. (13) demonstrated that disruption of DsbA, the enzyme that catalyzes disulfide bond formation, and disruption of NlpE’s C-terminal domain disulfide bond resulted in activation of CpxAR. This suggests that the C-terminal domain of NlpE may have an additional role in sensing disulfide bond formation (13). In addition, the *dsbA* gene is a Cpx regulon member, so this sensing would establish a neat feedback loop. Taking the data together, May et al. (7), in parallel with Delhaye et al. (13), have expanded the idea of the role of NlpE as a sensor for multiple stimuli in E. coli; the N-terminal domain of NlpE senses defects in lipoprotein maturation and transport, while the C-terminal domain senses cell adhesion to hydrophobic surfaces (11, 12) and defects in disulfide bond formation (13).

These exciting findings from May et al. expand on the mechanism by which high levels of copper are toxic to Gram-negative bacteria. While copper has been best described for its role in generating reactive oxygen species and disrupting iron-sulfur clusters in the cytoplasm (19), it is becoming increasingly evident that high levels of copper have adverse effects on the cell envelope that contribute to toxicity. Copper toxicity has been associated with membrane damage, and this was suggested to occur through lipid peroxidation (20), a mechanism demonstrated in yeast (19). However, lipid peroxidation occurs through reaction of copper ions with polyunsaturated fatty acids and bacteria typically produce only monounsaturated fatty acids (19). Instead, periplasmic copper accumulation could culminate in cell lysis by affecting multiple cell envelope biogenesis processes. Through reactions with Cys thiols, copper has been demonstrated to inhibit the ld-transpeptidase enzymes that remodel the peptidoglycan cell wall (21). While ld-transpeptidases are not essential, they form 3-3 cross-links that are critical for dealing with stress when LPS transport to the OM is reduced (22). Further, periplasmic copper can disrupt disulfide bond formation or catalyze incorrect disulfide bonds (23). The disulfide bond isomerase DsbC was demonstrated to be critical for correcting deleterious disulfide bonds introduced under conditions of copper stress (23). Importantly, the Lpt complexes of E. coli and many related pathogens contain an LptD subunit with two essential disulfide bonds that are rearranged by DsbC (24, 25). Finally, May et al. have provided evidence that high levels of periplasmic copper inhibit lipoprotein maturation, which would have downstream effects on OM biogenesis since each of the OM assembly complexes (Lpt, Bam, and Lol) contains lipoprotein subunits (2–4). Taking the data together, it is becoming increasingly clear that accumulation of copper in the periplasm disrupts assembly and transport of LPS, OMPs, and lipoproteins to the OM, thereby weakening the cell envelope, and that copper stress further disrupts the peptidoglycan remodeling that is critical when the OM is weakened.

The many facets of the process by which copper stress affects the Gram-negative cell envelope could explain the potent antimicrobial nature of copper surfaces (19). Knowledge about copper stress and the NlpE sensor could have important therapeutic implications. Copper and other metal ions are common chemicals used by the mammalian immune system to clear pathogens (26). Therefore, the NlpE/CpxAR stress response could have a critical role in resisting clearance by the immune system. In addition, the NlpE/CpxAR stress response provides protection against the antibiotic globomycin and compound 2, a promising inhibitor of lipoprotein transport. Understanding copper stress and the NlpE/CpxAR stress response could lead to new therapeutic strategies to target the Gram-negative cell envelope through the use of antimicrobials or to make pathogenic bacteria more sensitive to clearance by the immune system.
